# Novel optical gyroscope: proof of principle demonstration and future scope

**DOI:** 10.1038/srep34634

**Published:** 2016-10-03

**Authors:** Shailesh Srivastava, Shreesha Rao D. S., Hari Nandakumar

**Affiliations:** 1 Department of Physics, Sri Sathya Sai Institute of Higher Learning, Prasanthi Nilayam - 515134, A. P., India

## Abstract

We report the first proof-of-principle demonstration of the resonant optical gyroscope with reflector that we have recently proposed. The device is very different from traditional optical gyroscopes since it uses the inherent coupling between the clockwise and counterclockwise propagating waves to sense the rotation. Our demonstration confirms our theoretical analysis and simulations. We also demonstrate a novel method of biasing the gyroscope using orthogonal polarization states. The simplicity of the structure and the readout method, the theoretically predicted high sensitivities (better than 0.001 deg/hr), and the possibility of further performance enhancement using a related laser based active device, all have immense potential for attracting fresh research and technological initiatives.

Fiber optic gyroscopes (FOGs) and He-Ne ring laser gyroscopes (RLGs) have a distinguished record of being amongst the most sensitive and widely used optical sensors to date^1^. Millions of dollars have been pumped into research and development of fiber optic gyroscopes (FOGs) due to their extreme performance capabilities in critical applications^2^. The FOG technology has matured over the past two decades and a wide variety of physical effects have been controlled and utilized in the process. It is only the interferometric FOG (IFOG) that has distinctly reached high performances required in the strategic grade market with sensitivities better than 0.001 deg/hr. In this domain the IFOG is likely to dominate in the discernible future^1^.

In spite of the apparent maturity of a technology, there are many factors that lead to continued efforts to improve the existing technologies: scientific hunger for defeating the limits of performance, emergence of even more demanding applications hitherto not thought of, and competition from upcoming technologies to name a few.

In the tactical grade market, FOGs already have an upcoming competitor^3^ in micro-electro-mechanical-systems (MEMS) based devices, when it comes to precision guidance for machine control and general navigation systems. A high end ring laser gyroscope (RLG) was recently used for detecting the wobble of the earth, so as to aid in seismology and astronomical observations^4^. Its own twin technology, i.e. the ring resonator FOGs (RFOG) can achieve similar performances with fiber lengths that are two to three orders of magnitude less, however the inherent complexity and cost are the chief constraints which preclude the RFOGs commercial deployment^1^. It is evident that the need to push the limits of performance, to reduce the size, weight, complexity and overall cost of existing fiber optical gyroscopes, will continue.

In this scenario, it would be wise to think of novel ways to use the Sagnac effect^5^,^6^,^7^, or even use other mechanisms^8^, rather than only improve the existing methods.

We have recently suggested through theory and analysis^9^, a new way to use the Sagnac effect, which can turn out to be more cost effective and is also expected to perform better. This is the simplest possible coupled cavity device that consists of just a single ring and an inline reflector. This structure has the potential to be fabricated in the integrated optic form, as well as in the all-fiber optic form. This resonant optical gyroscope with reflector, deliberately utilizes the coupling between the CW and CCW waves to detect rotation, in contrast to other optical gyroscopes that avoid such coupling. The readout mechanism is definitely much simpler than that used in traditional RFOGs or RLGs, since rotation produces proportional power changes. The device has most of the advantages of both the IFOG and the RFOG.

In this paper we present a proof-of–principle demonstration of this device. Given our theoretical analysis and the demonstration presented here and with adequate research impetus, we believe this gyroscope has by and large, the potential to outperform the traditional RFOGs and IFOGs.

## Results and Methods

### Principal of New Gyroscope

**Figure 1:**
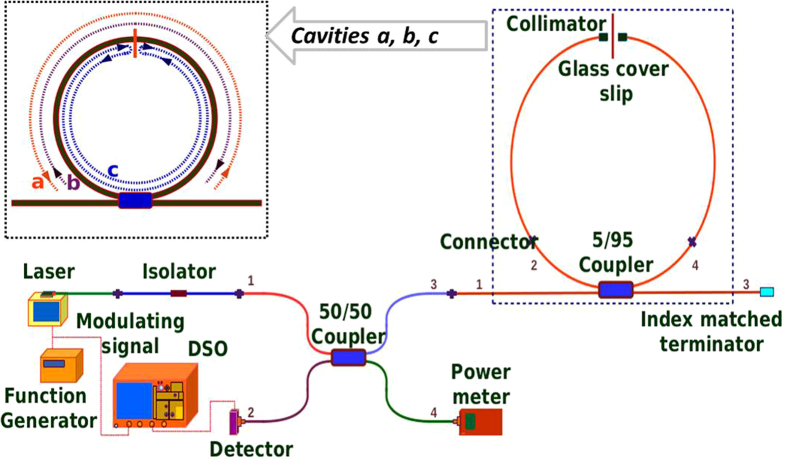
Experimental Setup. Inset (top left) shows pictorial representation of cavities (**a–c**).

To aid readability, the single coupler resonator with reflector (SCRWR) is shown in the dotted rectangle of Fig. 1. As described in detail in our theoretical work^9^, due to the presence of the inline reflector, there are three cavities, cavity **a**, with propagation in the CCW direction, cavity **b,** with propagation in the CW direction and cavity **c** with propagation equally in both these directions. Cavity **c** is formed due to reflections from the two sides of the reflecting element and has a round trip path length that is twice that of **a,** or **b**. The flow paths of these cavities are shown pictorially in the dotted inset, on the top left of Fig. 1.

Detailed analysis of the cavities based on signal flow graphs, is published in our earlier work^10^. The signal flow paths show that the round trip amplitude transfer function of cavities are 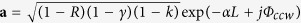, 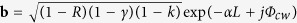, and **c** = *R*(1 − *γ*)(1 − *k*) exp(−2*αL* + *j*(*Φ*_*cw*_ + *Φ*_*ccw*_ + *π*)) = −*R***ab**/(1 − *R*), where *R* is the reflectance of the embedded reflector, *γ* is the coupling intensity loss coefficient of the 2 × 2 coupler, *k* is the intensity coupling coefficient of the coupler, *α* is the amplitude loss coefficient of the fiber, *L* is the round trip length of the ring and 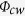 and *Φ*_*ccw*_ are the net round trip phase change of the clockwise and counter clockwise waves respectively. The cavity **c** is anti-resonant at the resonance of **a** or **b** due to the extra phase of π when the ring is stationary. This can cause mode splitting even when the cavity is at rest, provided *R* is larger than a critical value^9^ *R*_*crit*_ = (*k*/(2 − *k*))^2^. When the ring rotates clockwise the resonance of **a** and **b** split as *Φ*_*cw*_ = *Φ* + Δ*Φ* and *Φ*_*ccw*_ = *Φ* − Δ*Φ* due to the Sagnac effect. We note that the resonant frequency of **c** remains unaffected by rotation. Application of the Mason’s rule to the signal flow graph of the resonator^10^, gives the amplitude reflection coefficient of the ring as 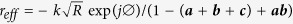 so that the intensity reflectance of the ring is *R*_*eff*_ = |*r*_*eff*_|^2^. This gives us


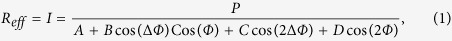


where *P, A, B, C* and *D* are constants that depend on the values of the coupling coefficient, the reflectivity of the inline reflector and the losses^9^. Equation (1) with *Φ* = 0 gives us the change in the peak reflectance (*R*_*eff*_ for *Φ* = 0) with rotational velocity and is used for sensing.

**Figure 2:**
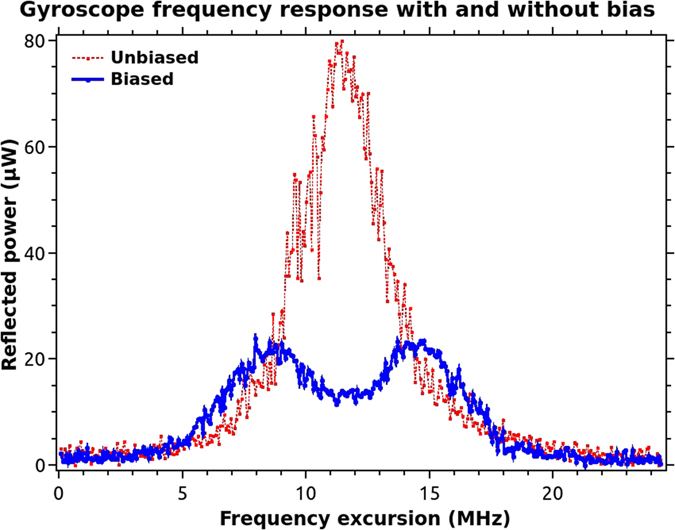
Principle of rotation sensing. The bigger peak is without rotation and the smaller split peak is due to an equivalent rotational velocity introduced by adding a nonreciprocal phase bias to the cavity (explained in a later section in the text).

Figure 2 shows the reduction and splitting of the resonant peak due to rotation, where the horizontal axis corresponds to the round trip phase *Φ* in terms of frequency change. It is interesting that the plot of *R*_*eff*_ versus Δ*Φ* follows a curve very similar to the shape of the big peak in Fig. 2, with Δ*Φ* = 0 at the center. This implies that the maximum sensitivity (max. of d*R*_*eff*_/dΔ*Φ*) is around the half maximum point where *R*_*eff*_ = *R*_*eff*_ (peak)/2. The gyroscope will need to be biased at this point (Δ*Φ*_*b*_) using some nonreciprocal phase bias mechanism. We also note that the sensitivity for the unbiased stationary gyroscope (Δ*Φ* = 0) is zero. We note that the non-reciprocal bias in Fig. 2 is more than the optimum value, so *R*_*eff*_ is below the half maximum point. This plot was generated using very large nonreciprocal bias of about 0.96 rad, which in our case is equivalent to a rotational velocity of about 9.5 rad/sec and this was used done only to demonstrate the principle. This was achieved using two quarter wave plates as explained later and was the first idea to be experimentally verified.

We have earlier^9^ derived the expression for the minimum detectable rotation rate under lossless conditions. This can be recast in the form


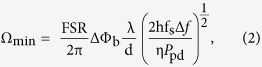


where P_pd_ is the average power on the photodetector (I_o_/2) and Δf is the detection bandwidth, f_s_ is the resonant frequency, η is the quantum efficiency of the photodetector and d is the diameter of the ring.

The standard expression for the ring resonator fiber optic gyroscope^2^ (RFOG) differs from this expression only by replacement of the factor (FSR/2π)ΔΦ_b_ by the FWHM of the resonator Δν. We note that (FSR/2π)ΔΦ_b_ is the effective HWHM of the plot of *R*_*eff*_ versus Δ*Φ* expressed in frequency units. As seen earlier^9^, this effective HWHM is smaller than the HWHM of the resonator (Δν/2). Therefore theoretically the minimum detectable rotation rate is smaller than half that of the RFOG without reflector under ideal conditions for the same FWHM of the resonator. So the sensitivity is twice as good.

Using our formula for finesse^10^ of lossless reflecting resonator, F = π/[R_crit_ (R_crit_ + 2)]^1/2^, we see that achieving large finesse greater than 100 (small FWHM) is not easy. We need extremely small embedded reflectances, where the degradation due to loss becomes the limitation.

However the advantage in our structure is that power can be significantly increased without degradation due to nonlinear Kerr effect as we have seen in our simulations^9^. This is because the CW and CCW waves are already coupled in our cavity. This has a two-fold effect as the resonator length can now be increased to decrease FWHM and increase sensitivity. We do not need to control the power difference of two counter propagating waves and should not require sophisticated frequency shifters/phase modulators to produce the beat frequency as in RFOGs. Overall we have the possibility of sensitivities comparable and better than RFOG with cheaper and broader linewidth sources, similar resonator sizes and reduction in complexity^9^.

### Experimental setup

The experimental set up is shown in Fig. 1. The SCRWR was made using a 2 × 2 coupler with fiber arm lengths of 0.5 m each, and an intensity coupling ratio of 5/95 (AFW Technologies). Two fiber-pigtailed AR coated c-lens collimators (AFW Technologies), with fiber lengths of 1.5 m each, were added to the arms of the coupler, to give a total cavity length of 4 m. An ordinary cover slip made of glass, was used between the two collimators as the reflecting element. A single frequency narrow linewidth fiber laser (SFL1550P-Thorlabs) was used as the optical source. Due to the noise in the DC bias provided by the laser driver (CLD1015-Thorlabs), the linewidth of the laser was broadened to about 300 kHz from its rated minimum of 50 kHz. An isolator was used after the laser to avoid back-reflection. The laser output was coupled to the SCRWR using a 50/50 coupler (Thorlabs) and this allowed the reflected output of the SCRWR to be monitored using a photo-detector. All the fibers arms and the 2 × 2 couplers in the entire set up consisted of polarization maintaining fiber (PMF). Output from the laser was linearly polarized and all fiber connectors were the FC/APC type, with their fast axis aligned to the key. The c-lens collimators used within the cavity had a working distance of 10 cm.

The reflected power was measured from the output arm (arm 2) of the 50/50 coupler using a fast response, low noise, biased germanium detector (DET50B/M-Thorlabs). The detector had a fiber coupled input to minimize coupling losses. The output of the photo detector was fed to a Stanford Research Systems low-noise pre-amplifier (SR560-SRS) and then observed on a digital oscilloscope (DL9140-Yokogawa). The detector was calibrated before the experiment, and this way the absolute optical intensity on the photo-detector could be known.

The resonant peaks of the SCRWR were observed by sweeping the laser frequency. This was achieved by modulating the laser drive current with a periodic ramp voltage using a function generator (DS345- SRS). The 20 mV p-p ramp caused negligible change in the laser power, but was sufficient to frequency modulate the laser over 324 MHz. Since the cavity length of 4 m had an FSR of about 50 MHz, the laser was being swept over a little more than six FSRs. Using the laser specifications this corresponded to a wavelength sweep from 1550.9187 nm to 1550.9213 nm.

All average power measurements were done using a fiber pigtailed power meter (VTC 830). The reflectance of the cover slip was measured and kept at 2.8%. This value could be varied by minor adjustment of the cover slip. The total round trip loss that included all the coupler losses, as well as insertion losses due to the collimator and reflector-assembly was measured to be about 31%.

An improvised smooth rotational platform was constructed using an optical bread board (Holmarc). Due care was taken to avoid other sources of vibration by mounting the set up on suitable vibration isolation supports. The rotational velocity in this setup was recorded using a smartphone application (Physics Toolbox Suite) that could measure rotational velocity as small as 0.01 rad/sec (~2000 deg/hr). The stage was rotated with a varying angular velocity up to about 4–5 radians per second.

Since sophisticated phase modulators to control and feedback stabilize the SCRWR cavity were not available, an innovative approach was used for detecting the rotation. This was based on the knowledge that in the absence of other perturbations, the peak reflectivity of the SCRWR gyro depends only on the rotation rate.

### Proof-of-principle sensing

**Figure 3:**
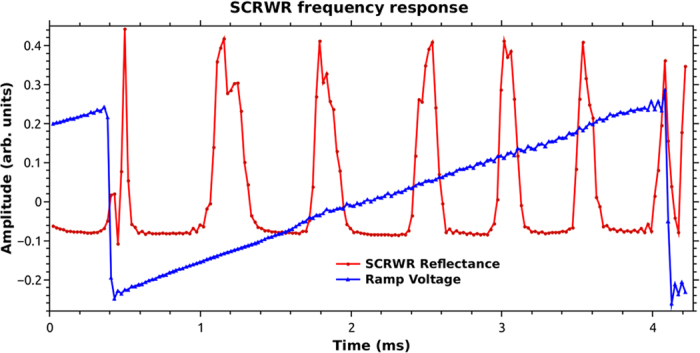
Frequency response of the SCRWR.

For the purpose of sensing, the output of the detector was fed into one channel of a 16 bit stereo audio card and sampled at 44100 samples per second. The modulation voltage ramp that was used to sweep the frequency of the laser was fed to the second channel of the audio card. As the entire set up was rotated, the data captured by the audio card (Fig. 3) was recorded on a PC, while the mobile phone app also recorded the rotational velocity.

The ramp frequency driving the laser was kept at 270 Hz, which corresponds to a time period of about 3.7 msec. The photon lifetime in the SCRWR cavity was only a few round trip times, due to the large losses. This would be hundreds of nanoseconds at most. Thus the SCRWR response, as traced on the DSO was approximately the same as the expected steady state response for the measured experimental parameters. In Fig. 3, the decreasing width as well as spacing of the peaks along the ramp was found to be due to the non-linear frequency change of the laser diode with drive current. The finesse of the resonator was about 6.25 by simulations under our experimental conditions. As the rotation velocity increases to large values the splitting causes the effective finesse to reduce further. The experimental value as in Fig. 3 is about 4 and is smaller due to the higher rotational velocity and a partly due to the fast ramp. Ramp frequency much above 270 Hz was found to broaden the peaks considerably as well as lower the peak values. The analysis of the various noise sources that change the peak reflectivity is presented in a later section of this paper. Other features of the peaks in this figure are explained under that section.

**Figure 4:**
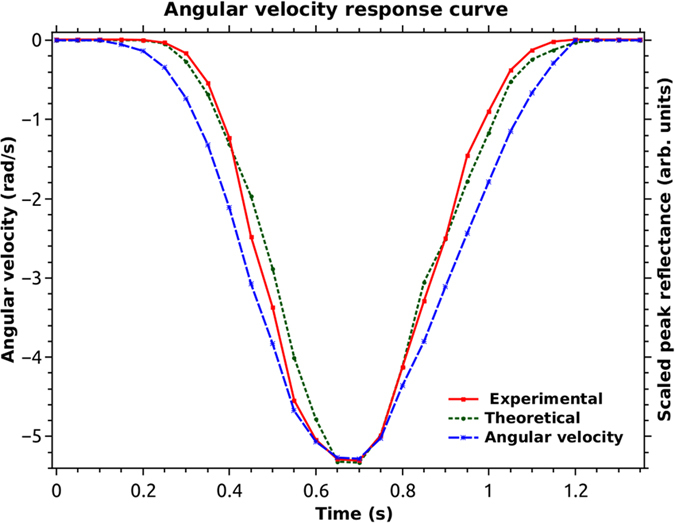
Sensing rotation using the unbiased gyroscope.

The rotation table was given a unidirectional rotation velocity for a period of about one second, while the SCRWR was made to sweep across its response curve. There are more than five peaks in one ramp period of 3.7 milliseconds, so we had more than 1000 peak points within the one second duration of rotation. A program was written in Scilab, to read the recorded wave file and extract these peak values in synchronization with the ramp waveform. This ensured that the global time variable was the same for all the plots, including the data from the smartphone application. The curve that resulted from these peaks, had noise due to the vibration of the bulk optic reflecting element, as well as noise due to the Fabry-Perot type cavity formed between the SCRWR and the Fresnel reflection at the output fiber arm 3. This plot was further smoothened using a digital low pass filter to obtain the solid plot in Fig. 4. An input power of 10.7 mW was launched into the resonator and the peak reflectance for this experiment was measured to be about 0.0175 without rotation and this value decreased to about 0.005 as the rotational velocity increased to above 5 rad/sec.

The theoretically expected reflected peak intensities corresponding to the rotational velocity curve obtained from the smartphone application were also plotted (dotted plot) using equation (1). It can be seen that the experimental and the theoretical values fit well within the proof-of-principle objective of this demonstration.

### Biasing the gyroscope for enhanced sensitivity and direction discrimination

As we pointed out earlier the sensitivity of the SCRWR-gyro varies with the bias point. In particular the sensitivity is zero for the unbiased (stationary) case. This is also evident in Fig. 4, where we note that till almost 0.5 rad/sec (10^5^ deg/hr) the change in output is near zero, implying zero sensitivity.

It is clear that to achieve maximum sensitivity the gyroscope needs to have an inherent non reciprocal phase difference between the counter propagating fields.

For a typical cavity with radius of a few cm to a few meters it requires a very large rotational velocity of to reach the point of maximum sensitivity. This could be achieved using a non-reciprocal element in the cavity. We propose here, a novel way to achieve this without any magneto optic elements. If the counter propagating fields could be made orthogonally polarized, the inherent birefringence of the polarization maintaining fiber could be used to provide the required phase difference. In what follows, we give a proof of principle demonstration of this biased gyro using quarter wave plates.

In our bulk optic set up for the reflecting element, we introduced two AR coated quarter wave plates (QWP) (Thorlabs), one on either side of the reflector. The fast axis of one QWP was kept at 45° to the fiber Eigen axis while the other QWP had its slow axis along this direction. This way the light which gets transmitted through the glass slip and both the wave plates, remains unchanged in its state of polarization. The light that gets reflected from the reflecting element back through the same QWP becomes polarized orthogonal to the incident light wave. The cavity **a,** and cavity **b** as discussed earlier, have light waves going in counter clockwise and clockwise directions and now these are orthogonally polarized states. Cavity **c** has half the round trip in one state of polarization and the other half orthogonally polarized to the first one. The birefringence of the PMF making up the ring resonator produces a 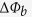 that can be kept at the point of maximum sensitivity, by choosing a PMF fiber length L that is an appropriate fraction of its beat length L_b_. Since the nonreciprocal phase difference repeats periodically after every beat length, one can select L = mL_b_ + ΔL_b_, where m is an integer and ΔL_b_ is the desired fraction of L_b_. This could produce any ΔΦ_b_ between 0 and 2π. The same could also be achieved by temperature tuning the birefringence.

The added advantage of biasing is that we can now distinguish between the two directions of rotations. In one case the rotation induced phase difference *ΔΦ*_*rot*_ adds to the existing Δ*Φ*_*b*_, while in the other case the sign of Δ*Φ*_*rot*_ is opposite to the existing bias. In the former case the peak reflectance decreases further, while in the latter it increases.

**Figure 5:**
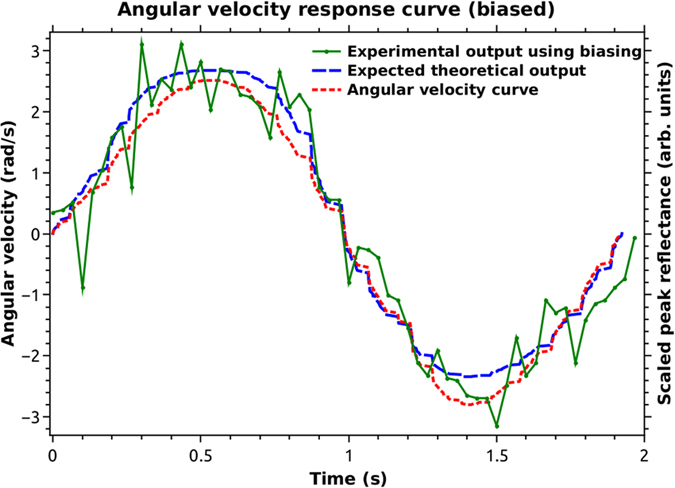
Sensing rotation using the biased gyroscope.

**Figure 6:**
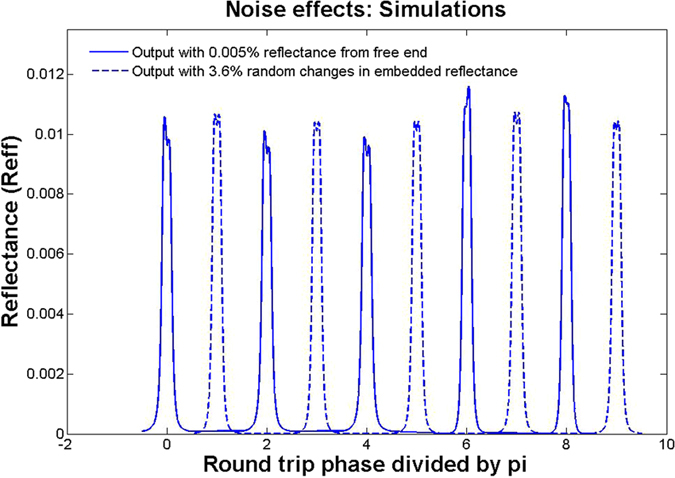
Effect of noise using simulations. Dashed plot is output variation due to vibration induced misalignment of embedded reflector causing a 3.6% change in its effective reflectance at a rotational velocity of 2.8 rad/sec. Solid plot is output variation due to finite reflectance (0.005%) from the free fiber end, with its phase varying over 0 to 2π for the same rotational velocity. (Dashed plot is horizontally offset only for clarity).

**Figure 7:**
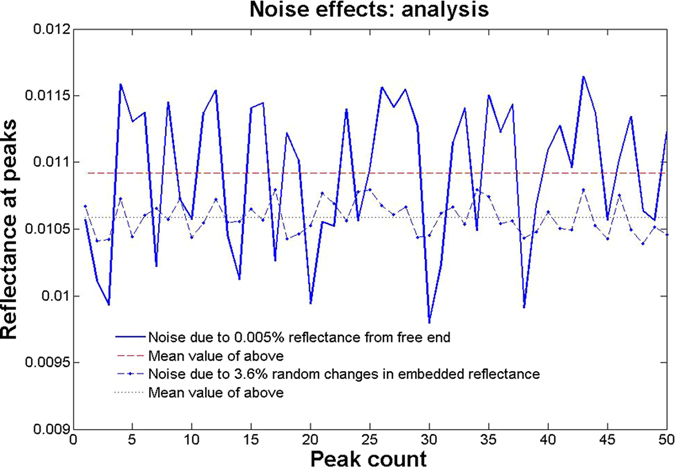
Effect of Noise. The values of peaks in Fig. 6 have been extracted and plotted to clearly show the fluctuations and mean value over 50 peak counts.

Figure 5 shows the experimental result (noisy solid plot) with the bulk optic QWPs, used to provide a bias Δ*Φ*_*b*_, such that without rotation, the output peak reflectance was about half that without the QWPs. The dotted plot is the velocity of rotation from the smartphone application. These velocity values were given as input to our simulation which was based on a complete polarization based analysis of the SCRWR gyro. The output of the simulation gave us the dashed plot. Other experimental conditions were Δ*Φ*_*b*_ = 0.28 *radians*, an experimental R_eff_ of about 0.0107 without rotation, which compares very well with the simulated value of 0.0109 (Figs 6 and 7) using all measured experimental parameters in equation (1). Since the bias point was slightly above the half-maximum point (since R_eff_ at 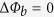 was 0.018), the velocity curve in Fig. 5 is slightly flat for positive angular rotation velocities above 2 rad/sec. We noted that the AR coated QWPs did not introduce any measurable loss and the net reflected power was measured as 113.5 μW, while the launched power was again 10.7 mW. All conditions were otherwise experimentally identical to the unbiased case.

One can see that the output now goes in two opposite directions, depending on the sign of the angular velocity. The experimental output has not been filtered and is noisy. Within the scope of the experiment the output fits well to the expected theoretical values. We can now see large sensitivity even at zero angular velocity and the sensitivity matched the smartphone application’s 0.01 rad/sec.

The real application of this gyroscope is in the extremely low rotation rate sensing under shot noise limited performance as we have shown before^9^. In our experimental set up there was no rotational bias stabilization and the losses were extremely high due to the bulk optic parts. The effect of residual vibration and residual Fresnel reflection from the free fiber end could not be compensated due to the nature of the set up. This made it impossible to sense small rotations. In an all fiber set up these sources of noise can be eliminated and with some standard feedback control of the nonreciprocal bias conditions, it will be possible to achieve shot noise limited performances. We discuss below the chief sources of noise and some ways to overcome these while making a real device.

## Discussion

### Sources of noise and their consequences

Since the rotation is sensed by measurement of back reflected peak powers, the sources of noise identified in order of their importance were Fabry-Perot effect due to the finite reflectance of the free end of the 2 × 2 coupler immersed in index matching liquid, change of effective reflectance of the embedded reflector (glass cover slip) due to vibrations, and thermal drift of nonreciprocal bias point due to temperature induced changes in birefringence. The laser output power was relatively constant compared to noise created by the above sources, as it was monitored and could easily be accounted for. With such complex relationship between various noise sources and outputs, simulations were the best way to explain the results and account for the experimental observations.

It was found that a very small reflectance from the free end, due to the index difference of the fiber core and paraffin could cause large variation in the peak reflectance of the gyro if the phase of the free arm varied from 0 to 2π. In the simulations, we have taken a uniform random distribution for this phase noise over 0 to 2π. A reflectance of 5 × 10^−5^ was taken considering the refractive index differences. The asymmetric random splitting observed experimentally in Fig. 3 was a result of this noise as can be seen from simulation results in Fig. 6. We show in Fig. 7, the extracted peaks from the plot of Fig. 6, over 50 such random peaks. The mean (upper straight line in the plot) and standard deviation of the final reflectance of the gyro were about 0.01095 and 0.00053 (*i.e*. ≈ ±5%). All other parameters in the simulation were as in the experiment.

The fluctuations in the effective reflectance of the embedded reflector were produced due to vibrations in the post on which the cover slip was held, as well as direct vibrations of the cover slip as it had to be kept freely hanging downwards, for want of space. Such flexural vibrations can typically be in the 100–1000 Hz range, and could therefore cause random fluctuations within one ramp period of 3.7 ms. The pendulum like vibration would cause a tilt of the hanging cover slip, which would cause considerable misalignment in the back reflection to the fiber at a distance of about 3 cm. This effect could be observed by measuring the back reflected power from one cavity arm, with the other arm blocked to disable the resonant effects. The dot-dashed curve in the Fig. 6 was produced using a 3.6% change (i.e. *R* = 0.028 ± 0.001) in the back reflected power due to vibrations. This plot was offset along the horizontal axis by 1/π radians per sec., for visual clarity. We can see that even such a large uniformly distributed random change produces a much smaller variation compared to the Fabry-Perot effect. The mean (lower straight line in Fig. 7) and standard deviation are 0.0106 and 0.0001 (*i.e*. ≈ ±1%) respectively. We note that the mean value is systematically lower without the averaged contribution from the Fresnel reflection at the free end. Figure 6 also shows that this noise does not produce the asymmetry in the split peaks that was experimentally observed in Fig. 3. We also point out that the solid plot in Fig. 7 has some correlation with the dot-dashed curve since it was generated using both the above random noise sources taken together. However, it was interesting to know using simulations that even when the embedded reflector’s vibration noise was taken to be zero, the Fabry-Perot type noise due to the free end produced the same standard deviation and splitting.

One way to reduce the Fabry-Perot type noise is to keep the free arm length as short as possible, use angle cut and better index matching and thermally insulate the arm. In our proof-of principle set-up this was unfortunately not possible. Since our gyro had high losses and small reflectance, the effect of the weak Fabry-Perot type end reflector was very strong. We have seen using simulations that this noise could also be reduced by lowering the losses in the ring. Active feedback control of this phase noise will also be useful.

The drift of the bias due to temperature induced changes in birefringence was found to be very slow and was well controlled by thermally insulating the resonator. If not controlled, this effect will also lead to changes in the peak reflectance. This noise source helped us in changing the bias but did not affect our observations.

### Future Scope

Most of the other limitations encountered can be overcome in an all-fiber set up. For this we can use in place of the two QWPs, two small pieces of PMFs, whose lengths are a quarter of the polarisation beat length and splice them at ±45° to the fast axis of the main fiber in the cavity. The embedded reflecting element could be created inline by coating the interface of the two pieces with TiO_2_ before fusion splicing^11^. Another method to create an inline reflector could be using femtosecond micromachining^12^ to create a refractive index discontinuity. It is important to note that if a nonreciprocal magneto optic method is used for biasing, the birefringence variation with temperature should cause no change since the CW and CCW waves would not be orthogonally polarised.

We already showed^9^ that our device only requires sources with linewidths that are an order of magnitude more than that used in RFOGs to achieve the same sensitivities. Even more promising are our preliminary simulations which show that if the SCRWR is used as an end reflector in a laser cavity as detailed in another earlier work^10^, we get a novel laser gyroscope whose sensitivity is dramatically increased. In this case the narrow linewidth laser would also not be required, and the rotation dependent average power output of the laser would be the readout. Much larger ring lengths (a chief limitation for RFOGs and RLGs) would only mean multimode oscillation and this would not be a problem since the power of each mode, would still change with rotation.

## Conclusion

In conclusion, we have given a first proof-of–principle demonstration of the newly proposed resonant optical gyroscope. A new technique for biasing the device has been proposed and experimentally demonstrated. The possibility of a laser gyroscope with even better performance has been revealed. We believe this is the begining of a fresh initiative in the research on optical gyroscopes and the future is promising.

## Additional Information

**How to cite this article**: Srivastava, S. *et al*. Novel optical gyroscope: proof of principle demonstration and future scope. *Sci. Rep*. **6**, 34634; doi: 10.1038/srep34634 (2016).
